# Supplementary material for: Bayesian refinement of protein structures and ensembles against SAXS data using molecular dynamics
Source: PLoS Comput Biol. 2017 Oct 18;13(10):e1005800. doi: 10.1371/journal.pcbi.1005800 (PMC5662244; doi:10.1371/journal.pcbi.1005800)
Supplement: S1 Archive — (BZ2) [file pcbi.1005800.s011.tar.bz2 › gromacs_4.62_bayesian_saxs_refi/share/html/online/g_order.html]

g\_order


| g\_order Main Table of Contents |

**VERSION 4.6  
Sat 19 Jan 2013**

---

### Description

Compute the order parameter per atom for carbon tails. For atom i the
vector i-1, i+1 is used together with an axis.
The index file should contain only the groups to be used for calculations,
with each group of equivalent carbons along the relevant acyl chain in its own
group. There should not be any generic groups (like System, Protein) in the index
file to avoid confusing the program (this is not relevant to tetrahedral order
parameters however, which only work for water anyway).

The program can also give all
diagonal elements of the order tensor and even calculate the deuterium
order parameter Scd (default). If the option -szonly is given, only one
order tensor component (specified by the -d option) is given and the
order parameter per slice is calculated as well. If -szonly is not
selected, all diagonal elements and the deuterium order parameter is
given.

The tetrahedrality order parameters can be determined
around an atom. Both angle an distance order parameters are calculated. See
P.-L. Chau and A.J. Hardwick, Mol. Phys., 93, (1998), 511-518.
for more details.  

### Files

| option | filename | type | description |
| --- | --- | --- | --- |
| **-f** | traj.xtc | Input | Trajectory: xtc trr trj gro g96 pdb cpt |
| **-n** | index.ndx | Input | Index file |
| **-nr** | index.ndx | Input | Index file |
| **-s** | topol.tpr | Input | Run input file: tpr tpb tpa |
| **-o** | order.xvg | Output | xvgr/xmgr file |
| **-od** | deuter.xvg | Output | xvgr/xmgr file |
| **-ob** | eiwit.pdb | Output | Protein data bank file |
| **-os** | sliced.xvg | Output | xvgr/xmgr file |
| **-Sg** | sg-ang.xvg | Output, Opt. | xvgr/xmgr file |
| **-Sk** | sk-dist.xvg | Output, Opt. | xvgr/xmgr file |
| **-Sgsl** | sg-ang-slice.xvg | Output, Opt. | xvgr/xmgr file |
| **-Sksl** | sk-dist-slice.xvg | Output, Opt. | xvgr/xmgr file |

### Other options

| option | type | default | description |
| --- | --- | --- | --- |
| **-[no]h** | bool | no | Print help info and quit || **-[no]version** | bool | no | Print version info and quit || **-nice** | int | 19 | Set the nicelevel || **-b** | time | 0 | First frame (ps) to read from trajectory || **-e** | time | 0 | Last frame (ps) to read from trajectory || **-dt** | time | 0 | Only use frame when t MOD dt = first time (ps) || **-[no]w** | bool | no | View output .xvg, .xpm, .eps and .pdb files || **-xvg** | enum | xmgrace | xvg plot formatting: xmgrace, xmgr or none || **-d** | enum | z | Direction of the normal on the membrane: z, x or y || **-sl** | int | 1 | Calculate order parameter as function of box length, dividing the box into this number of slices. || **-[no]szonly** | bool | no | Only give Sz element of order tensor. (axis can be specified with -d) || **-[no]unsat** | bool | no | Calculate order parameters for unsaturated carbons. Note that this cannot be mixed with normal order parameters. || **-[no]permolecule** | bool | no | Compute per-molecule Scd order parameters || **-[no]radial** | bool | no | Compute a radial membrane normal || **-[no]calcdist** | bool | no | Compute distance from a reference (currently defined only for radial and permolecule) |

---

http://www.gromacs.org  
gromacs@gromacs.org
